# Ophidiomycosis prevalence in Georgia’s Eastern Indigo Snake (*Drymarchon couperi*) populations

**DOI:** 10.1371/journal.pone.0218351

**Published:** 2019-06-12

**Authors:** Houston C. Chandler, Matthew C. Allender, Benjamin S. Stegenga, Ellen Haynes, Emilie Ospina, Dirk J. Stevenson

**Affiliations:** 1 The Orianne Society, Tiger, Georgia, United States of America; 2 Wildlife Epidemiology Laboratory, Department of Veterinary Clinical Medicine, College of Veterinary Medicine, University of Illinois Urbana-Champaign, Urbana, Illinois, United States of America; 3 Illinois Natural History Survey, Prairie Research Institute, University of Illinois Urbana-Champaign, Champaign, Illinois, United States of America; National Zoological Park, UNITED STATES

## Abstract

Wildlife diseases have posed a significant challenge to the conservation of many species in recent years. Diseases have been implicated in population declines over large geographic areas, with severe disease outbreaks leading to either local or complete extinctions of wild populations. Ophidiomycosis, commonly known as snake fungal disease, is caused by the fungus *Ophidiomyces ophiodiicola*, which has been documented in snake populations across the eastern and southern United States. We collected swab samples from the federally threatened Eastern Indigo Snake (*Drymarchon couperi*) in populations across the species’ Georgia range. We used quantitative PCR to determine the presence of *O*. *ophiodiicola* DNA and also recorded skin abnormalities characteristic of ophidiomycosis. From 1 September 2016 to 4 August 2018, Eastern Indigo Snakes tested positive for *O*. *ophiodiicola* DNA on 47 of 107 occasions (43.9%) and tested negative for fungal DNA but had skin lesions consistent with ophidiomycosis on 42 occasions (39.3%). Symptomatic and qPCR positive individuals were more likely to be encountered during January and February when compared to November and December. We found no effect of sex (p = 0.517), age-class (p = 0.106), or body size (snout-vent length: p = 0.083; mass: p = 0.206; body condition: p = 0.063) on ophidiomycosis status. Over the two-year study, we encountered individuals in which infection was clearly negatively impacting overall health and also documented individuals in which infection apparently cleared from one year to the next. These results demonstrate that *O*. *ophiodiicola* and lesions characteristic of ophidiomycosis are widespread in Georgia’s Eastern Indigo Snake populations. However, there are many unanswered questions regarding this disease, including the effects of disease on populations and individuals, the presence of infection vectors, and the change in prevalence over time. More research is needed to address ophidiomycosis and understand its impacts on ongoing conservation efforts.

## Introduction

Over the last three decades there has been an increased focus on wildlife diseases and the severe negative effects that they can have on individuals, populations, and even entire species or groups of species [1,2]. Population declines in frogs [3], salamanders [4], snakes [5], bats [6] and coral [7] have all been attributed, at least in part, to the effects of disease. Other stressors on wildlife populations can potentially increase disease susceptibility, creating interacting threats from which populations cannot recover [5,8]. In extreme cases, range-wide population declines have led to extinctions [9], although some species thought to be extinct have since been rediscovered [10,11]. As technological advances and anthropogenic activities continue to allow animals and microbes to travel easily around the globe, threats to wildlife from new and existing diseases are likely to increase.

The global decline of amphibian and reptile populations over the last century can be attributed to a variety of factors, and disease is often cited as a main contributor [3,12,13]. Many of the diseases negatively impacting herpetofauna are fungal pathogens that infect individuals through the skin [14,15]. A significant amount of research has been conducted on two fungal pathogens, *Batrachochytrium dendrobatidis* and *Batrachochytrium salamandrivorans*, which impact anuran and caudate populations, respectively [16–18]. Together, these fungi are responsible for the worldwide chytridiomycosis outbreak, which has resulted in local and global extinctions [3,19,20].

Diseases in reptile populations have generally not caused the precipitous population declines that have plagued amphibian populations in recent years. Reptilian diseases have therefore received less attention than their amphibian counterparts, although respiratory diseases in some tortoise populations have been well documented [21]. Recently, ophidiomycosis, sometimes referred to as snake fungal disease, has received increased attention as cases have been documented from at least 30 snake species from approximately 31 states across the eastern and southern United States [22]. Furthermore, the first case of ophidiomycosis in a European snake was recently documented [23], and it is likely that this disease exists in captive snake populations, potentially allowing it to spread worldwide. Ophidiomycosis has already been implicated in population declines of Timber Rattlesnakes (*Crotalus horridus*) in New Hampshire, where the disease was first documented [5].

Experimental infection studies have indicated that ophidiomycosis is caused by the fungus *Ophidiomyces ophiodiicola* [22,24]. *Ophidiomyces ophiodiicola* infects the dermal layer of snake skin, causing a variety of lesions that commonly manifest on the head and near the vent [15]. Prevalence of ophidiomycosis in snake populations can vary both spatially and temporally [25,26], and environmental conditions and stressors likely impact individual susceptibility to infection. Overall, little is known about ophidiomycosis prevalence, the environmental and biological factors that contribute to varying prevalence among species and over time, and the potential impacts of the disease on snake conservation.

Eastern Indigo Snakes (*Drymarchon couperi*) are listed as Federally Threatened under the U.S. Endangered Species Act [27] and listed as Threatened in both Georgia and Florida, where the vast majority of remaining populations exist today. Eastern Indigo Snake (hereafter EIS) populations throughout the species’ range have declined significantly in recent years, primarily because of habitat loss and degradation [28,29]. Today, populations are mostly restricted to large areas of protected Longleaf Pine (*Pinus palustris*) uplands and adjoining lowlands. In Georgia and northern Florida, EIS congregate on sandhills to breed in the late fall and early winter. Individuals largely remain in these habitats until warmer temperatures return in early spring, using Gopher Tortoise (*Gopherus polyphemus*) burrows to shelter from potentially lethal temperatures [30,31]. Today, EIS populations are still threatened by continuing habitat loss and degradation throughout the species’ range, even on protected lands [29,32]. Yearly long-distance movements in a region where development and the human population continue to expand also expose individuals to other threats, including roads, human persecution, and collection for the pet trade [28,33].

Here, we report on initial surveys documenting the prevalence of ophidiomycosis in EIS populations across southern Georgia, one of the remaining strongholds for this species. A single positive EIS was documented in Georgia in 2015, and recent surveys using the same methodology described below have documented *O*. *ophiodiicola* in over 20 other snake species in the state (H. Chandler, unpublished data). Our goal was to determine the prevalence of ophidiomycosis in EIS populations across their range in Georgia, particularly at sites where populations are currently protected.

## Materials and methods

### Study sites

**Fig 1:**
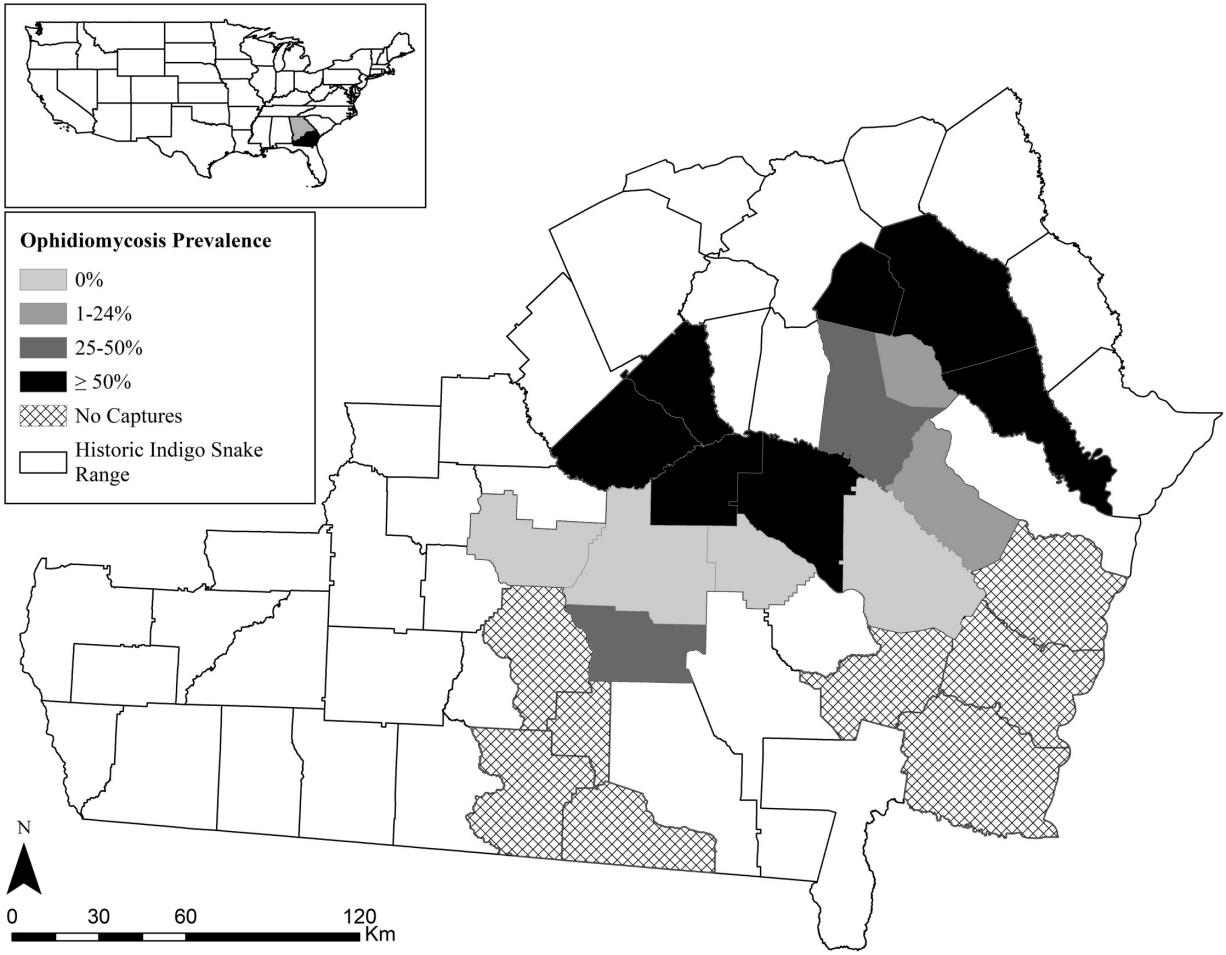
Georgia counties where Eastern Indigo Snakes (*Drymarchon couperi*) were surveyed for *Ophidiomyces ophiodiicola* and ophidiomycosis signs. Surveys were conducted from 1 September 2016 to 4 August 2018. The prevalence rate includes snakes that tested positive for *O*. *ophiodiicola* DNA using quantitative PCR.

We sampled EIS populations from the Coastal Plain of southern Georgia for *O*. *ophiodiicola*, surveying a combination of public and private lands. We targeted xeric sand ridges along three river drainages (Altamaha, Alapaha, and Satilla) with extant Gopher Tortoise populations, aiming to sample sites from across the species’ range in Georgia. In total, we surveyed for snakes on approximately 50 sites in 21 Georgia counties (Fig 1). However, EIS were not encountered at all sites that were surveyed, and it is likely that many of these sites are not currently occupied by EIS populations. We have withheld specific location information because of concerns about illegal collection of EIS.

### Sample collection

We began surveying snakes for *O*. *ophiodiicola* on 1 September 2016 and collected samples through 4 August 2018 (ca. 23 months). For EIS (S1 Fig), we focused survey effort from November to February when EIS are concentrated on sandhills with Gopher Tortoise burrows [31], although individuals were occasionally encountered outside of this time window. The majority of survey effort was devoted to visual encounter surveys. We targeted EIS by walking sandhills on days with air temperatures above 10° C and visually searching around Gopher Tortoise burrows for snakes [27,34]. A small number of EIS were encountered opportunistically during other surveys or while traveling to study sites. We captured all EIS by hand.

Upon capture, we visually inspected all snakes for skin lesions characteristic of ophidiomycosis [35]. After inspection, we collected swab samples from all individuals using a sterile cotton-tipped applicator. For individuals without lesions, we collected a single swab sample from the snake’s head. We gently, manually, restrained the snake’s head and applied firm pressure with the swab while rubbing the labial scales, nostrils, and chin. If an individual had skin lesions consistent with ophidiomycosis, we collected a head swab as well as additional swabs directly from the affected area(s). We placed all swab samples in 2.0 ml Eppendorf tubes and immediately froze samples at -20° until analysis. In addition to collecting swab samples, we sexed, weighed, and measured snout-vent-length (SVL) and total length for each individual. We classified individuals as juveniles, sub-adults, or adults following Stevenson et al. [36]. We also inserted a Passive Integrated Transponder (PIT) tag subcutaneously into each individual so that they could be identified if recaptured. We followed the United States Fish and Wildlife Service [37] guidelines when handling and processing EIS. We released all snakes near their point of capture, generally within an hour of capture. We used sterile handling procedures while collecting samples, including nitrile gloves changed between each snake, sanitizing hands between snakes, and cleaning equipment with an alcohol or bleach solution [38]. All sampling activities were approved by the United States Fish and Wildlife Service (Permit Number: TE28025A-2) and the Georgia Department of Natural Resources (Permit Numbers: 29–WJH–16–21, 029, and 115579244).

### Quantitative PCR

DNA extraction from swabs and quantitative PCR amplification (qPCR) from swabs were performed as previously reported [15]. DNA extraction followed the manufacturer’s recommendations with the addition of an incubation at 37°C with 25U of lyticase prior to the lysis step. Following DNA extraction, DNA quantity (measured in ng/ul) and quality (using the ratio of absorbance at 260 nm to 280 nm) were measured using spectrophotometry (Nanodrop, ThermoFisher Scientific). qPCR was performed in triplicate on a QuantStudio3 real time thermocycler. Samples were considered positive if replicates had a lower mean cycle threshold (C_t_) value than the lowest detected standard dilution. Copies per reaction were standardized to the total quantity of DNA in the sample by dividing the mean copies/ul for each sample by the DNA concentration, as determined by spectrophotometry.

### Statistical analysis

At the time of sample collection, all animals were assessed for clinical signs consistent with ophidiomycosis, including scabs, pustules, ulcers, necrotic areas, and/or displaced/thickened scales, and the presence or absence of clinical signs was recorded. Each snake’s clinical status was assigned based on the following classifications: 1) negative (no clinical signs, qPCR negative); 2) *Ophidiomyces* present (no clinical signs, qPCR positive); 3) possible ophidiomycosis (clinical signs present, qPCR negative); and 4) apparent ophidiomycosis (clinical signs present and qPCR positive). Body condition index (BCI) was calculated using the equation: Mass/SVL^2^ [39,40].

Fisher’s exact test was used to compare associations between ophidiomycosis classification and sex, age, month, season (Season 1: September 2016 to March 2017; Season 2: September 2017 to March 2018), and presence of lesions. We adjusted P-values using Hochberg’s sequential adjustment for multiple tests [41]. Logistic regression models were built to include all main effects and the covariates SVL and mass, but no interactions were considered due to sample size limitations. We then used an information theoretic approach to determine which model from our candidate set performed best using the AICcmodavg package [42]. Normality of continuous variables (SVL, mass, BCI) in the adult age class was assessed using the Shapiro-Wilk test. For normally distributed data, a one-way ANOVA and Tukey’s post hoc tests were used to evaluate differences within and between groups, respectively. For non-normally distributed data, a Kruskal Wallis ANOVA and Mann-Whitney U test were used to evaluate within and between group differences, respectively. To assess the association between presence/absence of skin lesions and qPCR status, sensitivity, specificity, positive predictive, and negative predictive values were calculated. All statistical analyses were performed in R or SPSS [43,44].

## Results

**Fig 2:**
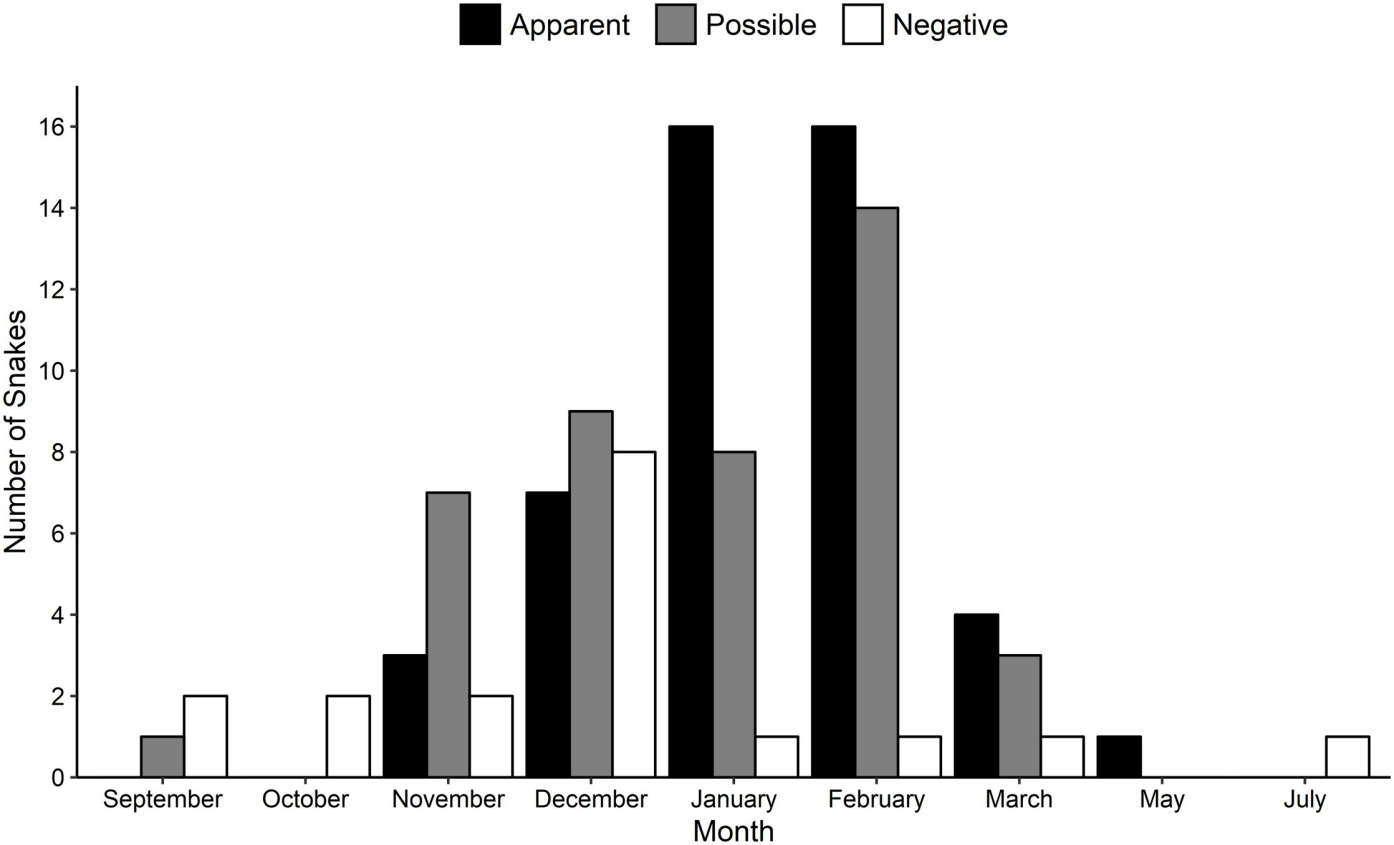
Seasonal ophidiomycosis prevalence in Eastern Indigo Snakes (*Drymarchon couperi*). Data were collected from 1 September 2016 to 4 August 2018 in southern Georgia. Snakes with apparent ophidiomycosis had skin lesions and were positive for *Ophidiomyces ophiodiicola* DNA using quantitative PCR. Possible ophidiomycosis was defined as snakes that had skin lesions but tested negative for *O*. *ophiodiicola* DNA. Negative individuals tested negative for *O*. *ophiodiicola* DNA and did not have any skin lesions.

Over the 2-year period, we collected 174 swab samples from 107 EIS encounters (Season 1: n = 44; Season 2: n = 61; plus two snakes caught outside of normal sampling seasons) (S1 Table). A total of 89 individuals were sampled, and 15 individuals were sampled on multiple occasions. Of these 89 snakes, there were 31 females, 49 males, and 9 snakes of unknown sex; 74 were adults, 10 were subadults, and 5 were juveniles (Table 1, S1 Table). Total length of sampled EIS ranged from 61.4–221.0 cm (mean = 170.4) and weight ranged 49–3674 g (mean = 1627). EIS were sampled mainly in December (n = 24), January (n = 25), and February (n = 31), but also, less commonly, in March, May, July, September, October, and November (Fig 2). We collected swab samples from EIS in 15 southern Georgia counties (Fig 1). All 15 counties had at least one individual with either apparent (11 counties, 73%) or possible (13 counties, 87%) ophidiomycosis. Out of the nine counties with more than five samples collected, four had a rate of apparent ophidiomycosis ≥ 50%.

**Table 1:** Number of sampling events and prevalence of ophidiomycosis in Eastern Indigo Snakes (*Drymarchon couperi*) sampled in southern Georgia from 2016–2018.

Variable	N	Negative	Possible ophidiomycosis	Apparent ophidiomycosis
Sample Season				
2003Sept. 2016–March 2017	44	10 (0.23)	21 (0.48)	13 (0.29)
Sept. 2017–March 2018	61	7 (0.12)	21 (0.34)	33 (0.54)
Age Class				
Juvenile	5	3 (0.60)	1 (0.20)	1 (0.20)
Sub-adult	10	3 (0.30)	5 (0.50)	2 (0.20)
Adult	92	12 (0.13)	36 (0.39)	44 (0.48)
Sex				
Female	39	4 (0.10)	18 (0.46)	17 (0.44)
Male	59	12 (0.20)	20 (0.34)	27 (0.46)
Unknown	9	2 (0.22)	4 (0.44)	3 (0.33)
Skin Lesions				
No	18	18 (1.0)	0 (0.0)	0 (0.0)
Yes	89	0 (0.0)	43 (0.48)	46 (0.52)

Apparent ophidiomycosis was defined by a positive quantitative PCR test for fungal DNA and the presence of skin lesions. Individuals with possible ophidiomycosis had skin lesions but tested negative for fungal DNA on quantitative PCR. Negative individuals had no skin lesions and tested negative for fungal DNA.

Over the two-year period, EIS tested positive for *O*. *ophiodiicola* DNA on 47 of 107 occasions (43.9%). All 47 qPCR positive results were associated with snakes that had skin lesions. In addition to these positive individuals, another 42 EIS had skin lesions when encountered but tested negative for *O*. *ophiodiicola* DNA (39.3%). The remaining 18 encounters were of snakes without skin lesions that tested negative for *O*. *ophiodiicola* DNA (16.8%). Individuals with a positive qPCR result were identified in six of nine months where at least one snake was sampled including in every month with at least five individuals tested (Fig 2). EIS were most likely to test positive for *O*. *ophiodiicola* DNA or have lesions present during January and February (39% of sampled snakes had an apparent ophidiomycosis and an additional 57% had skin lesions). Despite the majority of sampling events occurring in January and February (52%), only two individuals with no clinical signs of disease were detected during these months (Fig 2).

**Fig 3:**
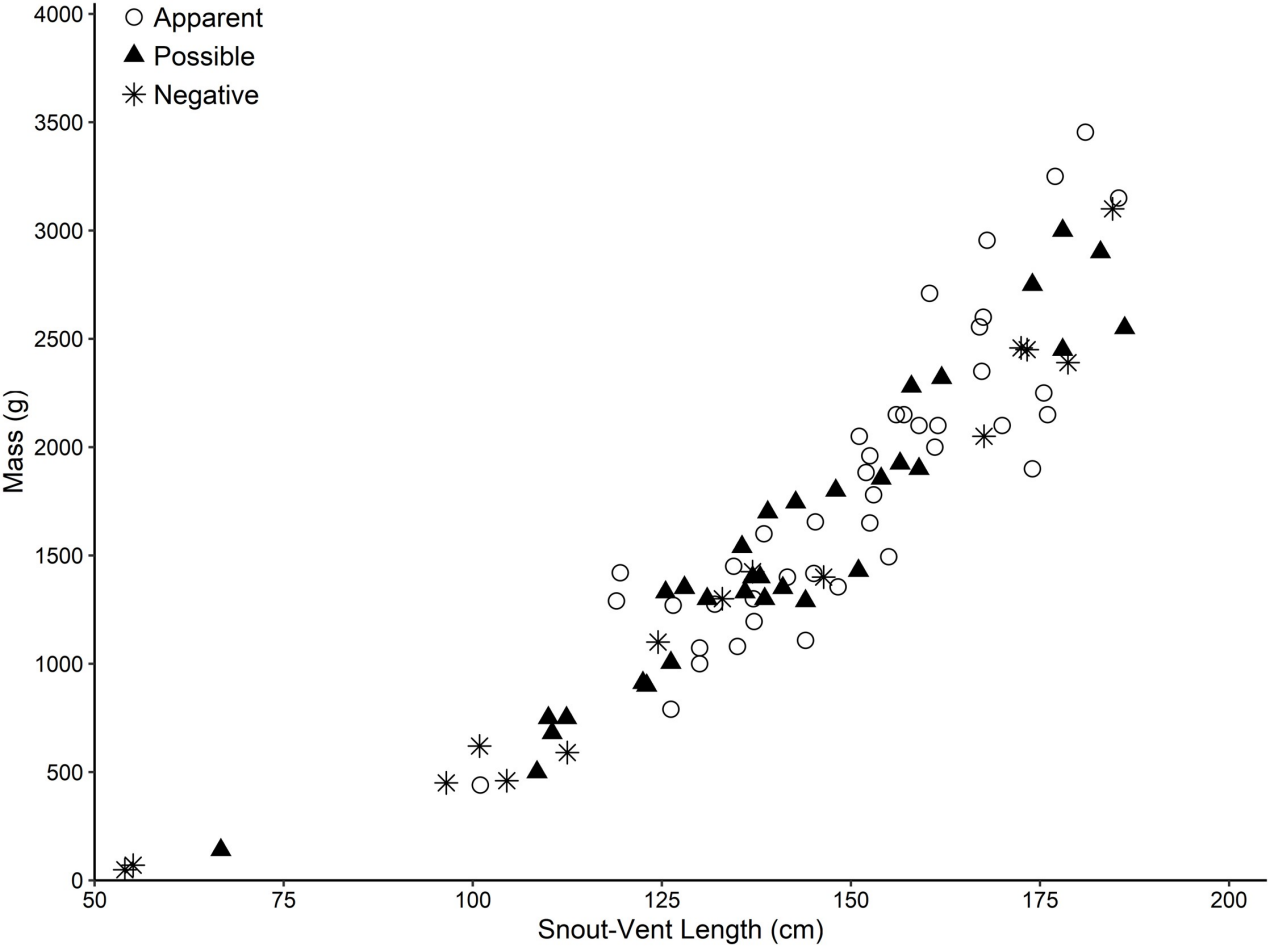
Ophidiomycosis prevalence in relation to body size in Eastern Indigo Snakes (*Drymarchon couperi*). All snakes were sampled in southern Georgia from 1 September 2016 to 4 August 2018 using cotton-tipped applicators. Snakes with apparent ophidiomycosis had skin lesions and were positive for *Ophidiomyces ophiodiicola* DNA using quantitative PCR. Possible ophidiomycosis was defined as snakes that had skin lesions but tested negative for *O*. *ophiodiicola* DNA. Negative individuals tested negative for *O*. *ophiodiicola* DNA and did not have any skin lesions.

Ophidiomycosis classification in EIS was significantly associated with month (p = 0.004) and presence of lesions (p = 0.0005) but not season (p = 0.106), age-class (p = 0.106), or sex (p = 0.517) (Table 1). The top logistic regression model included only the presence of lesions (AICcWt = 1.0) and thus univariate statistics are reported due to the apparent lack of statistical power in the multivariate approach. Skin lesions were nearly ubiquitous (present in 83.2% of encounters), and the sensitivity of skin lesions to detect apparent ophidiomycosis was 100%, the specificity was 27.7%, the positive predictive value was 49.5%, and the negative predictive value was 100%. Individuals did not significantly differ in SVL (p = 0.083), mass (p = 0.206), or BCI (p = 0.063) based on ophidiomycosis classification (Fig 3).

Fifteen individuals were sampled on multiple occasions. A majority of recapture events occurred within the same sampling season (40% recaptured across the two sampling seasons), and all 15 individuals were either positive for *O*. *ophiodiicola* DNA or had skin lesions at every capture event. Five individuals tested positive for *O*. *ophiodiicola* on multiple occasions, and two of these snakes tested positive a second time 11–13 months after the initial sampling event. Four individuals tested negative and then positive at a later date, while four other individuals tested negative at each sampling event. The remaining two individuals tested positive and then negative within the same sampling season. Finally, one additional individual tested positive in 2017 and was recaptured alive in 2018, but samples were not collected during the second capture event.

## Discussion

This is the first study to examine the prevalence of *O*. *ophiodiicola* and ophidiomycosis in EIS populations across Georgia, one of the species’ few remaining strongholds. The majority of snakes encountered in this study either tested positive for fungal DNA or had obvious skin lesions consistent with fungal infection. Furthermore, individuals testing positive for fungal DNA were identified from across the study area, and many of the populations that we surveyed are some of the largest remaining EIS populations, both in Georgia and range-wide [28]. Many of these populations exist on relatively well-maintained habitats with intact Gopher Tortoise populations and regular burn intervals [29,36]. High quality habitats can alleviate many of the threats to wildlife populations but not necessarily the impacts of disease [7,45,46].

The presence of skin lesions was the best predictor of ophidiomycosis classification in EIS, as all qPCR positive individuals had skin lesions. We documented a variety of different types of lesions on EIS during the two field seasons, including scabs, blisters, eroded and discolored scales, fluid-filled nodules, and other similar skin issues. Most of these lesion types have been reported from other species of snakes with ophidiomycosis [15,22,47,48]. Over the course of the study period, two individuals were captured that were almost completely covered with skin lesions from apparently severe infections. One of these individuals died while the other was successfully treated and released (T. Norton, personal communication). Other authors have previously remarked on the abundance of skin lesions in EIS populations in Georgia and Florida [36,49–52]. However, it remains unclear whether the lesions described in the literature are symptoms of ophidiomycosis or some other, more common, skin abnormality.

It is interesting to note that in EIS, the negative predictive value of skin lesions in identifying apparent ophidiomycosis was 100%. Conversely, the positive predictive value is poor, making it difficult to use clinical signs to confirm a diagnosis of apparent ophidiomycosis [53]. This is consistent with previous literature that showed the rate of false negatives is nearly 10 times higher in animals without lesions than individuals with lesions [48]. The current recommendation for sampling to reduce the false negative rate is to repeatedly and firmly swab along the entire surface of the skin up to eight times [48]. Our study was mostly conducted before these recommendations were published, and we elected to collect samples directly from skin lesions instead of swabbing along the entire length of the body. Lesions were most commonly located along the dorsal or ventral surface of the snake, below the head and above the vent. In many cases, multiple lesions along an individual’s body were swabbed. However, it is possible that our sampling methodology could lead to a higher rate of false negatives, especially in asymptomatic snakes where we only collected swabs from the head and not along the body [53]. The high number of snakes with lesions testing negative for *O*. *ophiodiicola* on qPCR also suggests that there are multiple causes of skin lesions in EIS.

There were temporal effects on infection rates and the presence of skin lesions. The majority of snakes with no signs of ophidiomycosis were captured from September–December, while apparent and possible ophidiomycosis cases were most common during January and February. These differences could be related to climatic and behavioral differences across a typical survey season. During cooler weather in late December and January, EIS in southern Georgia seldom feed and gradually lose body weight [36,54], potentially increasing their susceptibility to infections. Furthermore, by January and February, most EIS have spent 1–2 months in and out of Gopher Tortoise burrows, and the frequency of sheltering in tortoise burrows increases with lower winter temperatures [31]. Gopher Tortoise burrows are characterized by a stable temperature (approximately 15–18°C; [55]) and high humidity, which are conducive to *O*. *ophiodiicola* growth [15]. In fact, these environments are similar to the hibernacula utilized by Eastern Massasaugas (*Sistrurus catenatus*) and Timber Rattlesnakes, both of which have been significantly impacted by ophidiomycosis [5,25,48]. The propensity for Gopher Tortoise burrows to act as an ideal environment for fungal growth on EIS and other snake species utilizing these refuges, including Eastern Diamond-backed Rattlesnakes (*Crotalus adamanteus*), requires further study.

There was no significant impact of demographic parameters on ophidiomycosis classification. The majority of the snakes surveyed in our study were adults, and EIS less than 100 cm in SVL are rarely encountered in the wild [36,56]. However, several young-of-the-year EIS found at tortoise burrows during the winter have presented lesions similar to those seen in larger individuals (D. Stevenson, unpublished data). In general, there is little data available regarding ophidiomycosis in juvenile snakes. The impacts of infection on different age classes and sexes could potentially have important conservation consequences. For example, EIS population growth rate is more susceptible to changes in adult female survival when compared to other sexes or age classes [51].

We also failed to find a significant impact of ophidiomycosis on the body condition of infected individuals. In fact, most sampled individuals appeared to be of healthy weight with little indication that this disease or other skin infections were negatively impacting the individual’s overall condition, beyond the often ubiquitous skin lesions. Both ophidiomycosis and other fungal diseases have been shown to impact the behavior of infected individuals as they attempt to clear infections [25,57], and it is likely that EIS also respond to skin infections with behavioral changes. There are also other non-lethal impacts that fungal infections could have on individuals that would not have been apparent during our surveys, such as lower reproductive success. Overall, it remains unclear what effect, if any, *O*. *ophiodiicola* presence has on EIS populations in the wild. There is some evidence that *O*. *ophiodiicola* was present on at least one EIS from as far back as the 2004 (qPCR test, T. Norton, unpublished data), indicating that *O*. *ophiodiicola* may at least not be a recent development in EIS populations.

To date, there is still limited data on the natural course of ophidiomycosis in wild snakes and its effects at the population level. Complicating matters, many snake taxa are notoriously difficult to reliably survey in the field [58,59], and there is real concern about the negative impacts of surgically implanting radio transmitters in snakes, especially in populations with widespread disease [60] (J. Jensen and C. Jenkins, personal communication). EIS present an ideal opportunity to study the impacts of ophidiomycosis in wild snake populations because they can be reliably relocated each winter at tortoise burrows [36]. Several aspects of EIS natural history may also make them especially susceptible to fungal infections. In addition to overwintering in tortoise burrows, EIS have large home ranges and frequently consume other snake species [33,54], both of which could potentially expose them to *O*. *ophiodiicola*. Building long-term datasets that include population level infection rates over time, individual survival rates over time, a better understanding of infection vectors, hotspots of fungal growth (e.g., tortoise burrows), and the effects of fungal infection on snake behavior are crucial to fully understand and manage the impacts of ophidiomycosis.

## Conservation implications

Ophidiomycosis prevalence in EIS is concerning because populations have already declined across much of the EIS range and continue to face numerous threats, including habitat destruction, fragmentation, and degradation, the continued decline of Gopher Tortoise populations, climate change, and collection for the pet trade [61]. Effective conservation of EIS populations requires large tracts of undeveloped land, containing a mosaic of uplands and wetlands that allows snakes to use different habitat types throughout the year [62]. Our results indicate that, in southern Georgia, there are few, if any, EIS populations where *O*. *ophiodiicola* is not present. Recent survey data suggest that remaining EIS populations in this region have not experienced significant population declines from disease or other factors [29,36,51]. However, the increased percentage of infected individuals in our second survey season warrants additional monitoring to determine if ophidiomycosis prevalence is on the rise in these populations. Widespread disease has the potential to severely hamper conservation efforts for EIS, either through direct mortality, effects on behavior, or impacts on other life history traits. Expanded surveys are needed to assess *O*. *ophiodiicola* prevalence in Florida populations of EIS, particularly in southern Florida where EIS are less dependent on Gopher Tortoise burrows. There is growing concern that many snake species across the eastern and southern U.S. could be negatively affected by ophidiomycosis, and it will require a collaborative effort between biologists, veterinarians, and land managers to continue monitoring disease prevalence and its effects on wild snake populations.

## Supporting information

S1 Fig Adult Eastern Indigo Snake (*Drymarchon couperi*) from within the study area. (TIF)

S1 Table Capture, morphometric, and test result data for 107 Eastern Indigo Snakes (*Drymarchon couperi*) sampled for *Ophidiomyces ophiodiicola* and ophidiomycosis signs in Georgia. (XLSX)
