# Supplementary figures and images for: Ophidiomycosis prevalence in Georgia’s Eastern Indigo Snake (Drymarchon couperi) populations
Source: PLoS One. 2019 Jun 12;14(6):e0218351. doi: 10.1371/journal.pone.0218351 (PMC6561582; doi:10.1371/journal.pone.0218351)

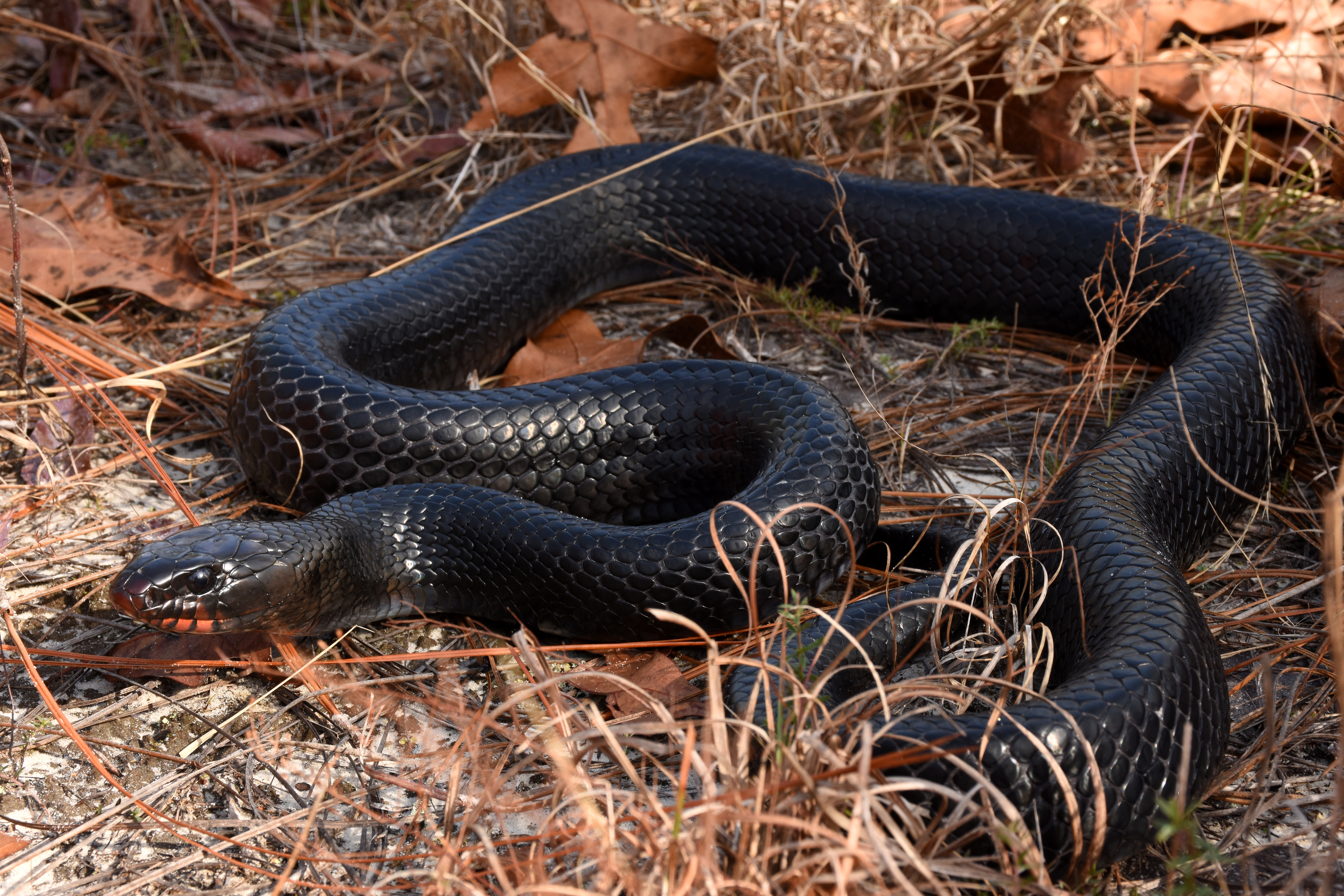

Supplement: S1 Fig — (TIF) [file pone.0218351.s001.tif]
